# Can narcissism be considered a risk factor for suicidal thoughts and behaviors? A systematic review of the literature

**DOI:** 10.1192/j.eurpsy.2021.1553

**Published:** 2021-08-13

**Authors:** V. Sprio, F. Madeddu, R. Calati

**Affiliations:** 1 Psychology, University of Milano-Bicocca, Milan, Italy; 2 Psychiatry, Nîmes University Hospital, Nîmes, France

**Keywords:** Narcissism, Suicidal risk, Systematic review, Suicide prevention

## Abstract

**Introduction:**

Although suicide showed an association with personality disorders, few studies focused on narcissism. This association is interesting, especially in what authors called a “narcissism epidemic”, considering narcissistic wounds to which subjects could be subjected.

**Objectives:**

To systematically review studies investigating the association between narcissism and suicidal risk.

**Methods:**

We focused on the association between narcissism (NPD, narcissistic traits) and suicide (Suicidal Ideation (SI), Non-suicidal Self-Injury (NSSI), Deliberate Self-Harm (DSH), Suicide Attempt (SA) and Suicide (S)). Studies were identified through a PubMed-based search. Reference lists were examined to extract additional articles. This review was performed according to PRISMA Statement.

**Results:**

We included 33 studies. Most studies evaluated narcissism through DSM, showing heterogeneous results. NPD was associated with low impulsivity and high planning, but also with a higher number of SAs. Studies evaluating narcissism as a trait were more coherent. SI, NSSI and DSH showed an association with vulnerable narcissism. These associations were explained by mediation and moderation models including shame and dissociation. The grandiose component was associated with severe repetitive NSSIs and S in high suicidal risk samples. Impulsivity showed no effect.

**Conclusions:**

It is possible to develop hypothesis, even if not causal relationships, on the association between narcissism and suicidal risk. Grandiose narcissism seemed to be protective for suicidal outcomes with low intent to die, while vulnerable narcissism seemed to be associated. However grandiose narcissism seemed to be a risk factor for suicidal outcomes with high intent to die, showing low impulsivity and high planning and severity. New studies, differentiating between narcissistic components, are needed.

